# Supplementary material for: Reconciling Mining with the Conservation of Cave Biodiversity: A Quantitative Baseline to Help Establish Conservation Priorities
Source: PLoS One. 2016 Dec 20;11(12):e0168348. doi: 10.1371/journal.pone.0168348 (PMC5173368; doi:10.1371/journal.pone.0168348)
Supplement: S1 Dataset — (ZIP) [file pone.0168348.s002.zip › Taxa/Serra Norte/SN_2013/N4E/Lista N4E-28.pdf]

CAVIDADE N4E-0028

Classe	Ordem	Fam/Outros	Gên/Outros	Espécie	S-U	U-S	Zon.
Arachnida	Acari	(Oribatida)		sp.3	X	X	P
Arachnida	Acari	Argasidae	*Ornithodoros*	sp.1		X	P
Arachnida	Acari	Mesostigmata		jovem		X	P
Arachnida	Acari	Trombiculidae		sp.		X	P
Arachnida	Acari	Trombidiidae	Microtrombidiinae	sp.		X	P
Arachnida	Amblypygi	Charinidae	*Charinus*	sp.1	1		P
Arachnida	Amblypygi	Phrynidae	*Heterophrynus*	*longicornis*	3	X	P
Arachnida	Araneae	Corinnidae		jovem	3	1	P
Arachnida	Araneae	Ctenidae		jovem		1	P
Arachnida	Araneae	Ochyroceratidae	*Speocera*	sp.1	X	X	P
Arachnida	Araneae	Oonopidae	aff. *Xyccarph*	sp.2	X	X	P
Arachnida	Araneae	Pholcidae	*Mesabolivar*	sp.	X	X	P
Arachnida	Araneae	Scytodidae	*Scytodes*	sp.		X	P
Arachnida	Araneae	Theridiidae	*Dipoena*	sp.3	X		P
Arachnida	Araneae	Theridiosomatidae	*Plato*	sp.		X	P
Arachnida	Opiliones	Stygnidae	*Protimesius*	*gracilis*	3		P
Arachnida	Pseudoscorpiones	Chernetidae	*Spelaeochernes*	sp.1	X	X	P
Arachnida	Pseudoscorpiones	Chthoniidae		sp.		X	P
Chilopoda	Scolopendromorpha	Cryptopidae	*Cryptops*	sp.		X	P
Chilopoda	Scutigeromorpha	Pselliodidae	*Sphendononema*	*guildingii*		X	P
Diplopoda	Polydesmida	Chelodesmidae		jovem	1	X	P
Diplopoda	Polydesmida	Pyrgodesmidae		jovem		X	P
Diplopoda	Spirostreptida	Pseudonannolenidae	*Pseudonannolene*	sp.1		X	P
Entognatha	Collembola	Paronellidae		sp.4	X		P
Entognatha	Collembola	Sminthuroidea		sp.		X	P
Entognatha	Diplura	Campodeidae		sp.1	X	X	P
Insecta	Blattodea	Blattidae		jovem	1	X	P
Insecta	Coleoptera	Tenebrionidae	Alleculinae	jovem	X	X	P
Insecta	Diptera	Chironomidae		sp.		X	P
Insecta	Diptera	Milichiidae		sp.		X	P
Insecta	Diptera	Psychodidae	Phlebotominae	sp.	X		P
Insecta	Diptera	Psychodidae	Psychodinae	sp.		X	P
Insecta	Diptera	Sciaridae		sp.		X	P
Insecta	Heteroptera	Cydnidae		jovem	X	X	P
Insecta	Homoptera	Cixiidae		sp.	X	X	P
Insecta	Hymenoptera	Formicidae	*Camponotus*	sp.		X	P
Insecta	Hymenoptera	Formicidae	*Hypoponera*	sp.		X	P
Insecta	Hymenoptera	Formicidae	*Pachycondyla*	sp.2	X	x	P
Insecta	Hymenoptera	Formicidae	*Prionopelta*	sp.1		X	P
Insecta	Hymenoptera	Formicidae	*Strumigenys*	sp.		X	P
Insecta	Isoptera	Termitidae	*Nasutitermes*	sp.	X		P
Insecta	Lepidoptera	Tineoidea		jovem		X	P
Insecta	Orthoptera	Phalangopsidae	*Phalangopsis*	sp.	22	X	P
Insecta	Psocoptera	Epipsocidae		jovem	X		P
Insecta	Thysanura	Nicoletiidae	Nicoletiinae	sp.1		X	P
Gastropoda	Pulmonata	Bulimulidae	*Naesiotus*	sp.	X		P
Gastropoda	Pulmonata	Subulinidae	*Allopeas*	sp.		X	P
Gastropoda	Pulmonata	Subulinidae	*Leptinaria*	sp.		X	P
Gastropoda	Pulmonata	Subulinidae		sp.4	X		P
Gastropoda	Pulmonata	Systrophiidae	*Happia*	sp.	X	X	P
Malacostraca	Isopoda	Scleropactidae	*Circoniscus*	*buckupi*	X	X	P
Amphibia	Anura			sp.	2		P
Mammalia	Chiroptera	Emballonuridae	*Peropteryx*	*kappleri*	3		P
Mammalia	Chiroptera	Furipteridae	*Furipterus*	*horrens*	2		P
Mammalia	Chiroptera	Phyllostomidae	*Carollia*	*perspicillata*	15		P

Mammalia	Chiroptera	Phyllostomidae		sp.		100	P
